# The associations between cardiometabolic risk factors and visceral fat measured by a new dual-energy X-ray absorptiometry-derived method in lean healthy Caucasian women

**DOI:** 10.1007/s12020-014-0180-7

**Published:** 2014-02-07

**Authors:** Tomasz Miazgowski, Barbara Krzyżanowska-Świniarska, Joanna Dziwura-Ogonowska, Krystyna Widecka

**Affiliations:** Department of Hypertension and Internal Medicine, Pomeranian Medical University, 71252 Szczecin, Poland

**Keywords:** Visceral fat, Dual-energy X-ray absorptiometry, Insulin resistance, Blood lipids

## Abstract

Excess visceral adipose tissue (VAT) is associated with a cluster of metabolic abnormalities. A new dual-energy X-ray absorptiometry (DXA)-based VAT measurement approach, CoreScan™, computes VAT mass and volume within the android region of a total body DXA scan. However, there have been no reference values developed for this method. The objective of this study was to determine the normal reference ranges for DXA-derived VAT in young, healthy, premenopausal women. We also sought associations between VAT, blood lipids, glucose, insulin and insulin resistance. In 120 randomly selected, normal weight, Caucasian women aged 20–40 years, we measured body fat (BF), VAT and lean mass by DXA. We also assessed blood pressure, waist and hip circumference, waist-to-hip ratio, body mass index, fasting glucose, insulin, triglycerides (TG), and high- (HDL) and low-density lipoproteins. Insulin resistance was evaluated by the homeostasis model assessment (HOMA). VAT mass accounted for 0.37 ± 0.3 % of weight and 1.11 ± 0.72 % of BF. Mean VAT mass and volume were 235.9 ± 183 g (95 % CI 202.7–269.1) and 250.3 ± 194.5 cm^3^ (95 % CI 215.1–285.4), respectively. Anthropometric indices moderately correlated with VAT. VAT significantly correlated with HDL (*R* = −0.193; *P* = 0.03), glucose (*R* = 0.252; *P* = 0.005) and HOMA (*R* = 0.184; *P* = 0.049). In this study, we provide normal values of VAT mass and volume measured by DXA and determined for healthy, normal weight, Caucasian women aged 20–40 years. Even in such strictly selected population VAT correlated positively with insulin resistance and inversely with HDL.

## Introduction

Visceral adipose tissue (VAT) accumulates primarily deep in the peritoneal cavity within the upper part of the abdomen and infiltrates the liver, stomach, pancreas and the mesentery. In contrast to abdominal subcutaneous adipose tissue (SAT), VAT is highly vascularized and contains large fat cells, which easily disrupt secreting proinflammatory cytokines, adipokines and hormones to the blood [1, 2]. It is also associated with impaired non-esterified fatty acid (NEFA) metabolism, leading to a hyperlipolytic state in which adipose cells are resistant to the antilipolytic effects of insulin. The resulting NEFA flux to the liver may induce increased hepatic production of glucose. Excess VAT is associated with a risk of type 2 diabetes, dyslipidemia, hypertension, cardiovascular diseases and cancer [3–5].

VAT may be assessed by surrogate methods, such as waist circumference (WC), waist-to-hip ratio (WHR), sagittal abdominal diameter and index of central obesity. It can be indirectly measured by a 3D full body scanner as body volume index or through bioelectric impedance analysis or dual-energy X-ray absorptiometry (DXA). Until recently, computed tomography (CT) and magnetic resonance imaging (MRI) have been the only widely used techniques for direct measurement of VAT mass and volume. However, these techniques are costly, time-consuming or associated with a risk of radiation. Earlier studies demonstrated the potential usefulness of DXA-derived indices to supply accurate measurements of VAT in addition to body composition [6, 7]. Recently, a new DXA-based VAT measurement approach, CoreScan, has been developed. The CoreScan algorithm computes VAT mass and volume within the android region of a total body DXA scan by subtracting SAT from total android fat. VAT computed by DXA has been validated using CT across a broad population of adults between 18 and 90 years of age and a wide range of BMI [8]. However, regardless of the method used for the assessment, there have been no reference values determined for VAT in healthy individuals and specific for gender, race and age. Therefore, in this study, we measured body composition in a homogenous group of young, healthy, premenopausal Caucasian women to determine the normal reference ranges for DXA-derived VAT. We also sought associations between VAT, blood lipids, glucose, insulin and insulin resistance in this population.

## Materials and methods

### Study population

Using local electoral lists, we randomly invited healthy women aged 20–40 years to participate in the study. The inclusion criteria included: BMI between 18.6 and 25.0 kg/m^2^, regular menstruation, no alcohol abuse and no medical conditions that required pharmacological treatment. We excluded women with a history of malignancy and prior hypertension, dyslipidemia, abnormal glucose tolerance (including gestational diabetes) and rapid weight changes (more than 3 kg) within the last 12 months. All subjects underwent a routine physical examination with a measurement of blood pressure in a supine position. Overall, of 247 women who participated in the study, we included 120 women who met the entry criteria.

This study complied fully with all applicable institutional and governmental regulations concerning the ethical use of human volunteers and with the terms of the Declaration of Helsinki. The Pomeranian Medical University Ethics Committee approved the study protocol, and all the recruited subjects gave their written informed consent.

### Anthropometric measurements

Height, WC and hip circumference were measured to the nearest 0.5 cm. WC was determined at the midpoint between the bottom of the rib cage and the iliac crest. Hip circumference was measured as the maximal circumference over the buttocks. From weight, height, WC and hip circumference, BMI and WHR were calculated. BMI was calculated as weight (kg) divided by height (m) squared. WHR was calculated as WC (cm) divided by hip circumference (cm) [9].

### Biochemical analyses

Biochemical analyses included fasting glucose, insulin and blood lipid levels. Serum glucose was measured after overnight fasting by the glucose oxidase method (Glucose Konelab/T Series, Fisher Scientific, Poland). Insulin was measured by the immunoenzymatic method (DPC Biermann GmbH, Bad Neuheim, Germany). From fasting glucose and insulin measurements, a HOMA-IR (homeostasis model assessment–insulin resistance) index was calculated. Serum total cholesterol, high-density lipoprotein cholesterol (HDL), low-density lipoprotein cholesterol (LDL) and TG were determined by enzymatic colorimetric methods using Roche Diagnostics assays.

### Measurements of visceral, android, gynoid and total fat

**Fig. 1:**
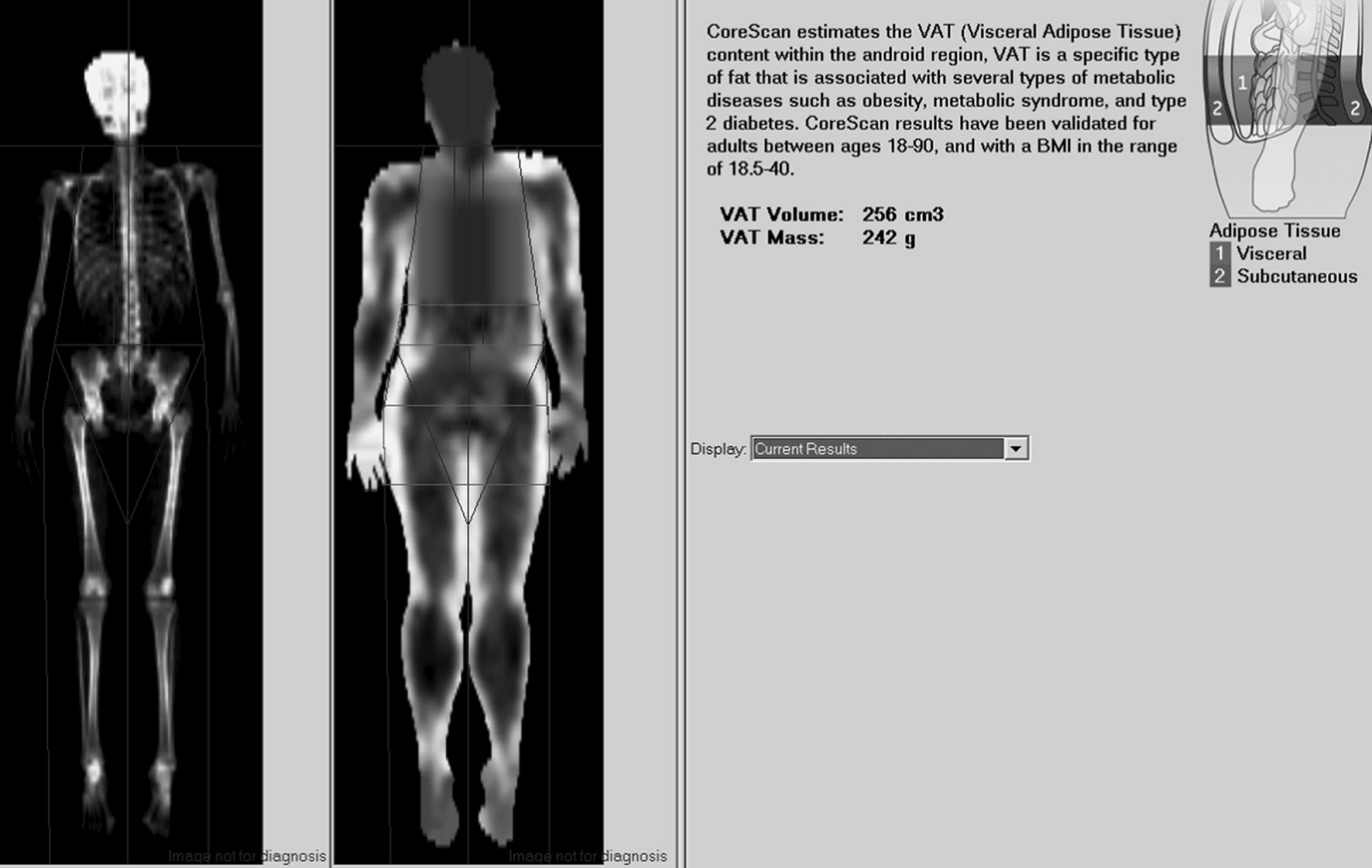
The assessment of visceral and SAT in the android region of interest by dual-energy X-ray absorptiometry. Total body scan of the study subject

Body composition, including total body fat (BF), regional fat (android, gynoid and visceral) and lean mass, was measured by DXA (GE Lunar Prodigy; Madison, WI, USA; 14.1 enCore software version with CoreScan™) using automatic total body scan mode. The regions of interest (ROI) for regional body composition were defined using the software provided by the manufacturer. The android region (Android), located over the abdomen, is roughly 10 cm in height, extending from the iliac crest toward the head for 20 % of the distance from the iliac crest to the base of the skull. The software algorithm works through detection of the width of the SAT layer on the lateral part of the abdomen and the anterior–posterior thickness of the abdomen, which can be determined using X-ray attenuation (Fig. 1). In the android ROI, we calculated SAT by subtracting VAT from android fat mass. The hip ROI (Gynoid) was defined superiorly below the pelvis cut line as 1.5 times the height of the android ROI, inferiorly below the superior line by two times the height of the Android ROI and laterally at the outer leg cut lines.

### Statistical analysis

Data are presented as mean ± standard deviation (95 % CI). Differences among the age bands (20–30 and 30–40 years) in normally and non-normally distributed variables were evaluated by Mann–Whitney *U* test or paired Student’s *t* test as appropriate. Because % BF, SAT, waist and hip circumference, WHR and Gynoid showed markedly skewed distributions, logarithmic transformations of these measurements were performed. Linear Spearman’s rank correlation coefficients were used to determine the relationship between body composition, anthropometric measurements and metabolic parameters. We used the 5 % significance for all tests.

## Results

Baseline characteristics are given in Table 1. Mean VAT/SAT ratio in the android ROI was 0.23 ± 0.14 %. VAT mass accounted for only 0.37 % of weight and 1.1 % of BF. Mean values of anthropometric measurements, blood pressure, serum glucose and insulin, and blood lipids were normal. When we analyzed the data in two age bands (data not shown), below (*n* = 40) and above 30 years of age (*n* = 80), we found no statistical differences in body composition, including VAT, except for a slightly higher VAT/SAT ratio in the older age band (0.18 ± 0.11 and 0.25 ± 0.15, respectively; *P* = 0.011).

**Table 1:** Baseline characteristics of study participants

	Mean	SD (95 % CI)
Age (years)	32.08	5.05 (31.2–33.0)
Weight (kg)	61.14	6.98 (59.9–62.4)
Height (cm)	166.55	5.69 (164.2–166.7)
Body mass index (kg/cm^2^)	22.23	1.82 (21.9–22.6)
WC (cm)	75.57	6.96 (74.3–76.8)
Hip circumference (cm)	95.72	5.94 (94.6–96.8)
Waist/hip ratio	0.79	0.05 (0.78–0.80)
Lean tissue (kg)	39.31	5.14 (38.4–40.2)
BF (kg)	19.70	4.32 (18.9–20.5)
BF (%)	31.75	4.69 (30.9–32.6)
Android fat (g)	1214.3	476.9 (1128.4–1300.2)
Gynoid fat (g)	3787.0	815.2 (3639.7–3934.3)
VAT (g)	235.93	183.4 (202.7–269.1)
VAT (cm^3^)	250.25	194.5 (215.1–285.4)
SAT (g)	978.38	336.1 (917.6–1039.1)
Visceral/SAT	0.23	0.14 (0.20–0.26)
VAT/BF (%)	1.11	0.72 (0.98–1.24)
VAT/Weight (%)	0.37	0.27 (0.32–0.42)
Glucose (mmol/L)	5.0	0.39 (4.94–5.1)
Insulin (μIU/mL)	6.65	3.3 (6.05–7.25)
HOMA	1.49	0.79 (1.35–1.63)
Total cholesterol (mmol/L)	4.82	0.93 (4.66–4.99)
Cholesterol HDL (mmol/L)	1.68	0.38 (1.61–1.75)
Cholesterol LDL (mmol/L)	2.74	0.82 (2.6–2.91)
TG (mmol/L)	0.83	0.47 (0.75–0.92)
Systolic blood pressure (mmHg)	116.7	14.01 (114.1–119.2)
Diastolic blood pressure (mmHg)	76.57	8.1 (74.7–78.3)

VAT was highly correlated with android fat (*R* = 0.832; *P* < 0.0001). Other correlations between body composition, anthropometric measurements and metabolic parameters are shown in Table 2. Anthropometric indices (WC, hip circumference, WHR and BMI) moderately correlated with VAT. Of these indices, BMI and WC were the most accurate measures of adiposity and regional fat depots. SAT, Android and BF showed weak but significant correlations with TG and HDL. Interestingly, VAT was also inversely associated with HDL level, but was the only measure that correlated with fasting glucose and HOMA. HDL (but not other metabolic parameters) inversely correlated with BMI (*R* = −0.208; *P* = 0.017) and WC (*R* = −0.226; *P* = 0.009). None of the studied parameters was associated with blood pressure values.

**Table 2:** Correlations between body composition, anthropometric measurements and metabolic parameters

	VAT (g)	VAT (cm^3^)	SAT (g)	Android (g)	Gynoid (g)	BF (g)	Waist (cm)	BMI (kg/m^2^)
Anthropometric measurements								
Waist (cm)	**0.645**	**0.642**	**0.703**	**0.752**	**0.571**	**0.722**		**0.717**
Hip (cm)	**0.336**	**0.338**	**0.535**	**0.522**	**0.683**	**0.613**	**0.598**	**0.571**
Waist/hip ratio	**0.535**	**0.531**	**0.398**	**0.477**	0.119	**0.365**	**0.713**	**0.400**
BMI (kg/m^2^)	**0.686**	**0.683**	**0.760**	**0.796**	**0.720**	**0.826**	**0.717**	
Metabolic parameters								
HDL (mmol/L)	**−0.193**	−**0.194**	−**0.198**	−**0.222**	−0.076	−**0.182**	−**0.226**	−**0.208**
LDL (mmol/L)	0.050	0.049	0.117	0.097	0.102	0.094	−0.075	−0.035
TG (mmol/L)	0.176	0.177	**0.251**	**0.254**	0.133	**0.214**	0.151	0.106
Insulin (μIU/mL)	0.138	0.140	0.075	0.097	0.107	0.120	0.006	0.023
Glucose (mmol/L)	**0.252**	**0.250**	0.018	0.096	0.002	0.076	0.014	0.029
HOMA	**0.184**	**0.179**	0.074	0.111	0.099	0.128	0.062	0.009
Systolic blood pressure (mmHg)	0.088	0.090	0.036	0.059	0.327	0.065	0.144	0.007
Diastolic blood pressure (mmHg)	0.122	0.120	0.079	0.101	0.022	0.097	0.171	0.051

Numbers in each cell describe Spearman’s rank correlation coefficient. Significant correlations are given in bold

## Discussion

In this study, for the first time, we have presented mean values and ranges of VAT assessed by DXA, determined in healthy, premenopausal Caucasian women. These data may serve as reference values for future research performed on similar populations. Increasing evidence suggests that VAT is an important measurement to make in assessing different phenotypes of obesity because its ectopic accumulation, particularly in the liver, is strongly associated with risk of metabolic diseases [5, 10]. However, until recently, routine use of this measurement has been limited by cost and access to CT or MRI, which have been the research tools for measuring VAT.

Previous research demonstrated the potential usefulness of DXA to supply accurate measurements of VAT. In 2000, Bertin et al. [7] compared DXA and anthropometric data and their combinations to the VAT area calculated from a CT scan. They found that the transverse internal diameter at umbilical level, which corresponded to the lean core of abdomen stripped of SAT, was the best predictor of VAT. Based on these findings, they developed an empiric equation to calculate VAT from height, sagittal diameter, and DXA-derived indices. Basically, the CoreScan algorithm is based on the similar assumption. It works through detection of two key parameters: (i) the width of the SAT layer on the lateral aspects of the abdomen and (ii) the anterior–posterior thickness of the abdomen, which can be attained using the DXA tissue attenuation image. A simple geometric model using these measures is used to estimate the android SAT. Therefore, the CoreScan application computes VAT by subtracting the android SAT from the total android fat. This new DXA software application provides a high degree of precision and accuracy in a single measurement [8–11]. Moreover, DXA is fast, safe and presents little discomfort or inconvenience to the subjects. DXA exposes subjects to a much lower radiation dose than CT (below 1.00 μSv for a standard-mode total body DXA exam vs. 3,100 μSV of radiation for an abdominal CT scan), making it suitable for repeated measurements. Finally, MRI or CT measurements predict cardiovascular risk in relation to VAT area or volume, while DXA additionally offers an assessment of VAT mass. The greatest utility for CoreScan would be to characterize cardiometabolic risk, not only as a fast screening tool in subjects at risk but also as a tool for assessment of therapeutic efficacy, as longitudinal assessment of body composition during controlled under- and overfeeding shows that small decreases and increases in fat mass are associated with corresponding decreases and increases in insulin secretion and insulin sensitivity [12]. Moreover, recent data suggest that visceral fat increases and decreases proportionately with short-term weight gain and loss [13].

Numerous clinical studies have documented significant associations between CT-derived VAT and metabolic diseases [14–18]. To our knowledge, only one clinical study has evaluated VAT by DXA in a healthy population. In a cross-sectional analysis of body composition across a wide range of age bands (25 subjects per each band) in healthy Italian blood donors, Bazzocchi et al. [19] demonstrated that VAT mass assessed by DXA was age- and gender-dependent; the mean VAT value in their study was 149 ± 156 g in females aged 18–30 years and 358 ± 326 g in those aged 31–40 years. Overall, their results regarding VAT are similar to ours, although there are differences between BMI, lean mass and SAT results in the two studies. This strongly suggests that future research is needed to determine cutoff values for DXA-derived VAT to identify subjects at risk for metabolic syndrome, as has been determined for VAT measured by CT [17]. Recently, in a large cohort of Polish women representing a wide range of BMI, we found that Android mass above a threshold value of 1,500 g increased the risk of metabolic syndrome by 2.07 times (95 % CI 1.05–4.09) [20]. Since in the present study Android constituted ~20 % of VAT, we can speculate that the VAT cutoff value for metabolic syndrome determined in healthy women of normal weight aged between 20 and 40 years would be about 300 g.

We also found that VAT was positively correlated with fasting glucose level and HOMA and negatively with HDL. These results suggest that even in healthy and lean individuals, excess VAT may be associated with a cluster of metabolic abnormalities, including insulin resistance, abnormal lipid profile and ectopic (e. g. epicardial) fat deposition [21, 22], which appear to be key factors in the pathogenesis of metabolic syndrome and metabolically obese but normal-weight phenotype. This underlines the importance of developing accurate methods for early quantification of VAT. Our results indicate that among the surrogate methods of measurement of total/abdominal obesity, WC and BMI better assess VAT than WHR or hip circumference. However, anthropometric variables were only moderately associated with VAT (*R* < 0.7), similarly as in earlier studies in which VAT was measured by CT [7]. Moreover, WC and BMI, in contrast to VAT, correlated only with HDL but not with other metabolic parameters.

When interpreting our data, it is appropriate to consider certain limitations of our study. The main limitation is its cross-sectional design. Hence, the associations presented between independent factors and outcome variables do not necessarily represent causal relationships. We presented data from a relatively large sample of healthy, premenopausal Polish women with narrow BMI, BF and age ranges. Therefore, data presented in this study might not be applicable for general populations or other ethnicities. A study that involves a broader range of age and BMI is important for further validation of these findings. Second, in addition to possible inter-operator variation, there is intra-equipment variation in DXA measurements, even within equipment supplied by the same manufacturer, which may influence the reliability of measurements in population-based studies. Moreover, in some cases, an automatic scan mode does not fix the markers accurately within the studied ROI, which requires manual corrections. To minimize such technical errors, during the entire study period, we used the same DXA scanner and software version. Additionally, all scans were analyzed by the same technician.

In conclusion, in this study, we provide normal values of VAT mass and volume measured by DXA and determined for healthy, normal weight, Caucasian women aged between 20 and 40 years. Even in such strictly selected population, VAT appears to predict the risk of insulin resistance and unfavorable lipid profile.
